# Value of the Application of CE-MRI Radiomics and Machine Learning in Preoperative Prediction of Sentinel Lymph Node Metastasis in Breast Cancer

**DOI:** 10.3389/fonc.2021.757111

**Published:** 2021-11-19

**Authors:** Yadi Zhu, Ling Yang, Hailin Shen

**Affiliations:** ^1^ Department of Radiology, The First Affiliated Hospital of Soochow University, Suzhou, China; ^2^ Department of Radiology, Suzhou Kowloon Hospital, Shanghai Jiaotong University School of Medicine, Suzhou, China

**Keywords:** breast cancer, radiomics, sentinel lymph node metastasis, machine learning, CE-MRI

## Abstract

**Purpose:**

To explore the value of machine learning model based on CE-MRI radiomic features in preoperative prediction of sentinel lymph node (SLN) metastasis of breast cancer.

**Methods:**

The clinical, pathological and MRI data of 177 patients with pathologically confirmed breast cancer (81 with SLN positive and 96 with SLN negative) and underwent conventional DCE-MRI before surgery in the First Affiliated Hospital of Soochow University from January 2015 to May 2021 were analyzed retrospectively. The samples were randomly divided into the training set (*n*=123) and validation set (*n*= 54) according to the ratio of 7:3. The radiomic features were derived from DCE-MRI phase 2 images, and 1,316 original eigenvectors are normalized by maximum and minimum normalization. The optimal feature filter and selection operator (LASSO) algorithm were used to obtain the optimal features. Five machine learning models of Support Vector Machine, Random Forest, Logistic Regression, Gradient Boosting Decision Tree, and Decision Tree were constructed based on the selected features. Radiomics signature and independent risk factors were incorporated to build a combined model. The receiver operating characteristic curve and area under the curve were used to evaluate the performance of the above models, and the accuracy, sensitivity, and specificity were calculated.

**Results:**

There is no significant difference between all clinical and histopathological variables in breast cancer patients with and without SLN metastasis (P >0.05), except tumor size and BI-RADS classification (P< 0.01). Thirteen features were obtained as optimal features for machine learning model construction. In the validation set, the AUC (0.86) of SVM was the highest among the five machine learning models. Meanwhile, the combined model showed better performance in sentinel lymph node metastasis (SLNM) prediction and achieved a higher AUC (0.88) in the validation set.

**Conclusions:**

We revealed the clinical value of machine learning models established based on CE-MRI radiomic features, providing a highly accurate, non-invasive, and convenient method for preoperative prediction of SLNM in breast cancer patients.

## Introduction

Breast cancer, the second leading cause of cancer-related death, has become the most commonly diagnosed cancer among women worldwide (1). In recent years, the incidence rate of breast cancer has been increasing, which seriously threatens women’s physical health and quality of life. Identifying axillary lymph node (ALN) status is essential for breast cancer patients because it has great significance for the breast cancer clinical stage, treatment plan, and prognosis of patients (2). The ALN status is also one of the important reference indexes for postoperative radiotherapy and chemotherapy (3). ALN dissection (ALND) has long been used to determine the status of ALN in patients with breast cancer. However, ALND is an invasive operation with some significant limitations, including infection, nerve damage, shoulder dysfunction, arm numbness, and upper limb lymphedema (4, 5). As the first lymph drainage station, the sentinel lymph node (SLN) metastasis status and numbers can predict the axillary node metastasis status, and help to decide to further axillary management or not (6). SLN biopsy (SLNB) is the recommended procedure for clinical evaluation of lymph nodes in tumor-free areas of breast cancer patients. However, it is still an invasive procedure with complications such as arm numbness or lymphedema in 3.5–10.9% of patients (5, 7). Moreover, the long intraoperative pathology waiting time prolongs the anesthesia time, thus reducing the efficiency of the surgery. Therefore, a non-invasive and precise diagnostic approach with higher clinical applicability is urgently needed for preoperative evaluation of sentinel lymph node metastasis (SLNM).

Imaging examinations such as ultrasonography, mammography, CT, and MRI are commonly used to diagnose breast cancer. However, these methods are difficult to estimate SLNM accurately and have high false-negatives. In recent years, with the rapid development of artificial intelligence, radiomics has drawn increased interest. Radiomics can convert digital medical images into high-dimensional, exploitable, and quantitative imaging features. These features can expose intratumor heterogeneity and provide potential non-invasive biomarkers for clinical-decision support (8–10). Machine learning algorithms are crucial for identifying and recognizing useful radiomic features related to outcome variables. Using different machine learning algorithms to construct predictive models and compare their performance can mine the best predictive models better (11–13).

In this study, contrast-enhanced MRI (CE-MRI) images were used for radiomics analysis, and a variety of machine learning algorithms were used to build predictive models, aiming to explore the value of CE-MRI radiomics and machine learning in preoperative prediction of SLNM in breast cancer.

## Materials and Methods

### Study Population

This retrospective study was approved by the Institutional Review Board and conducted under Good Clinical Practice guidelines. Informed consent was waived. Consecutive patients with histologically confirmed breast cancer between January 2015 and May 2021 were retrospectively reviewed. Inclusion criteria were as follows: (1) patients with breast cancer confirmed by histopathological examination, and (2) received SLNB/ALND; (3) patients underwent dynamic contrast-enhanced MRI examination before surgery; (4) available clinical and pathological information {such as age, tumor size, BI-RADS classification, histological type and grade of invasive carcinoma, molecular subtype [according to the status of estrogen receptor (ER), progesterone receptor (PR), human epidermal growth factor receptor type 2 (HER-2), and Ki-67]}. Exclusion criteria were as follows: (1) patients who underwent preoperative endocrine therapy, neoadjuvant chemotherapy, or radiotherapy; (2) ipsilateral breast surgery; (3) MRI examination data were incomplete, or image quality was poor. One hundred seventy-seven patients were enrolled in this study (177 lesions containing 81 SLN metastasis and 96 non-SLN metastasis).

### Pathological Evaluation

SLNB was performed for all patients after the MRI examination. Methylene blue tracer was used to identify the SLN during operation, then HE staining was performed on the resected SLN. The SLN was defined as metastatic when there were macro-metastases (malignant cell clusters larger than 2 mm) or micro-metastases (approximately 200 cells, larger than 0.2 mm, but none larger than 2.0 mm). The results were confirmed by two pathologists with more than 10 years of experience.

According to the criteria of the 2013 St Gallen International Expert Consensus (14), breast cancer was divided into four different molecular subtypes, namely, Luminal A: ER(+) and/or PR(+), HER-2(−), Ki-67≤14%; Luminal B: HER-2(−), ER(+) and/or PR(+), Ki-67≥14%, HER-2(+), ER(+) and/or PR(+), any Ki-67; HER-2 overexpression: ER(−), PR(−), HER-2(+); triple-negative breast cancer: ER(−), PR(−), HER-2 (−). Tumors with 10% or more immunostaining cells were considered ER or PR positive. The HER2 status was determined to be positive when the IHC staining intensity score was ≥3. Identification of gene amplification by fluorescence *in situ* hybridization (FISH) was considered when the HER2 immunohistochemical score was 2+.

### MR Image Acquisition

MRI scans were performed on a 3.0T MRI scanner (GE Discovery 750W) equipped with an eight-channel breast-dedicated coil. The patient was in the prone position with both breasts naturally dangling and suitably fixed in the coil. The MRI sequences included axial T1-weighted imaging, axial T2-weighted imaging, DWI, ADC, DCE-MRI, and sagittal contrast-enhanced imaging. Images from a T1-weighted fat-suppressed dynamic sequence using a 3D fast gradient echo sequence (VIBRANT 3D, TR = 4.32 ms, TE = 2.10 ms; flip angle=14°°, slice thickness=1.4 mm, slice gap= −0.7 mm, matrix size=512 ×512, FOV= 350 × 350 mm) were used in analysis. The contrast agent was injected intravenously (0.1 mmol/kg of Gd-DTPA-MBA), then followed by a 15 ml saline flush, both at a rate of 2.6 ml/s. After intravenous injection, continuous non-interval scans were performed in five phases, with a scan time for each phase of 61 s. In this study, the contrast between the tumor and the background was the largest in the second phase (61~122 s) images, so the second phase of dynamic contrast-enhanced (DCE) images were used as the experimental data.

### Radiomics Workflow

**Figure 1:**
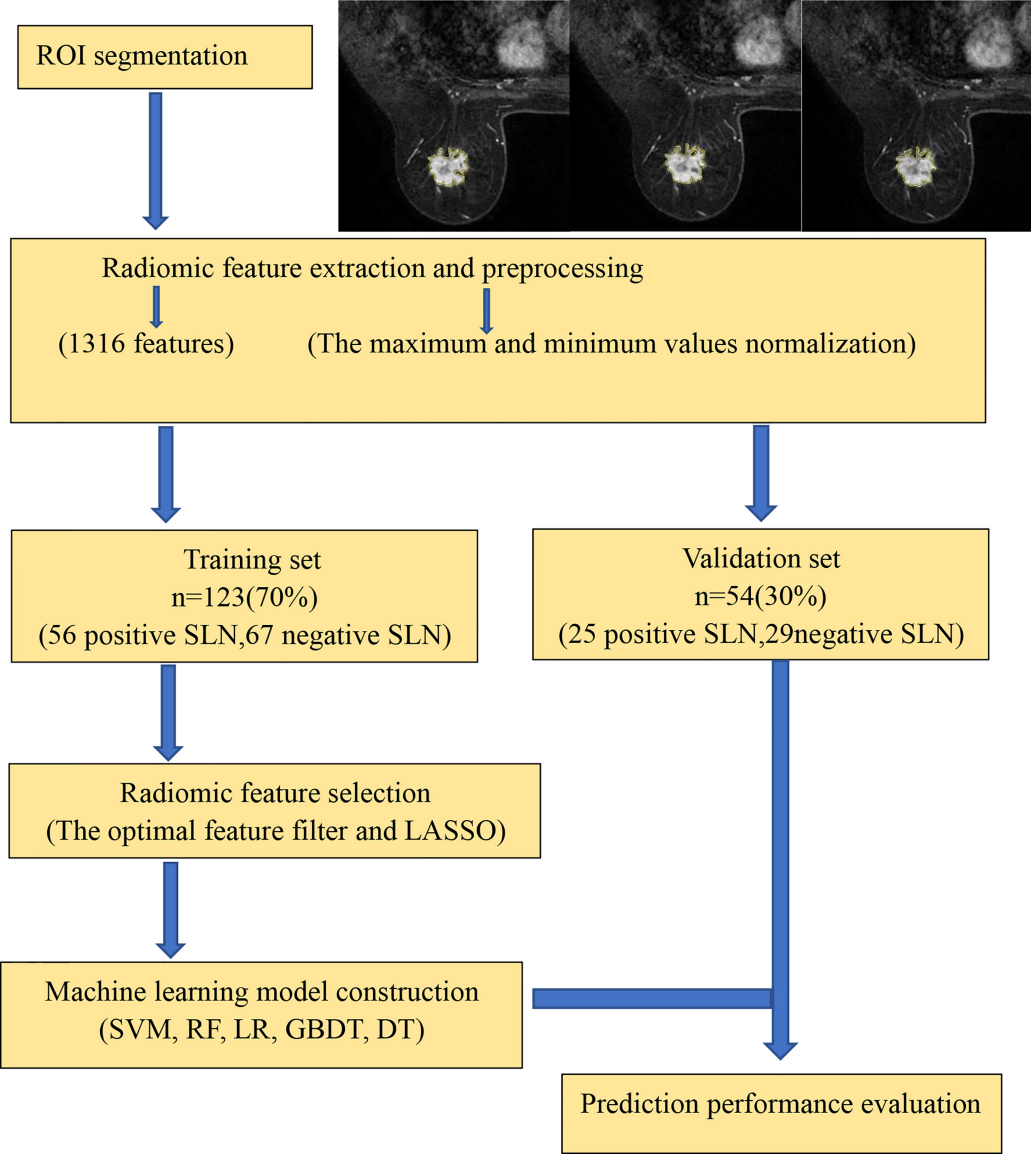
Radiomics workflow.

As shown in **Figure 1**, the prediction workflow includes (1) ROI segmentation, (2) radiomic feature extraction and preprocessing, (3) radiomic feature selection, and (4) machine learning model construction (using a training set) and prediction performance evaluation (using an independent validation set).

### Image Segmentation

Manual segmentation was performed on the axial second phase of T1-weighted images of dynamic contrast-enhanced (T1-DCE) images. The regions of interest (ROIs) were delineated using Darwin Scientific Research Platform (Beijing Yizhun Intelligent Technology Co., Ltd, China). The 3D-ROI was manually segmented by a radiologist with 5 years of experience who was blinded to the tumor’s histological type and the patient’s LN status, and all contours were reviewed by another senior radiologist with more than 10 years of experience. Cohen’s kappa method was used to assess inter-reader agreement. If the discrepancy was ≥5%, the tumor boundaries were determined by the senior radiologist.

### Radiomic Feature Extraction and Preprocessing

The platform mentioned above was used to extract radiomic features, a total of 1,316, including first-order statistics, shape-based 3D and texture features, etc. Texture features can describe the heterogeneity of the tumor, including Gray Level Dependence Matrix (GLDM), Gray Level Run Length Matrix (GLRLM), Gray Level Co-occurrence Matrix (GLCM), Gray Level Size Zone Matrix (GLSZM), and Neighboring Gray Tone Difference Matrix (NGTDM). For the classifier, data preprocessing can make the algorithm converge faster and obtain a more reasonable model. Therefore, the maximum and minimum values normalized are applied to linearly stretch the features of each dimension to the interval of [0,1].

### Feature Selection

**Figure 2:**
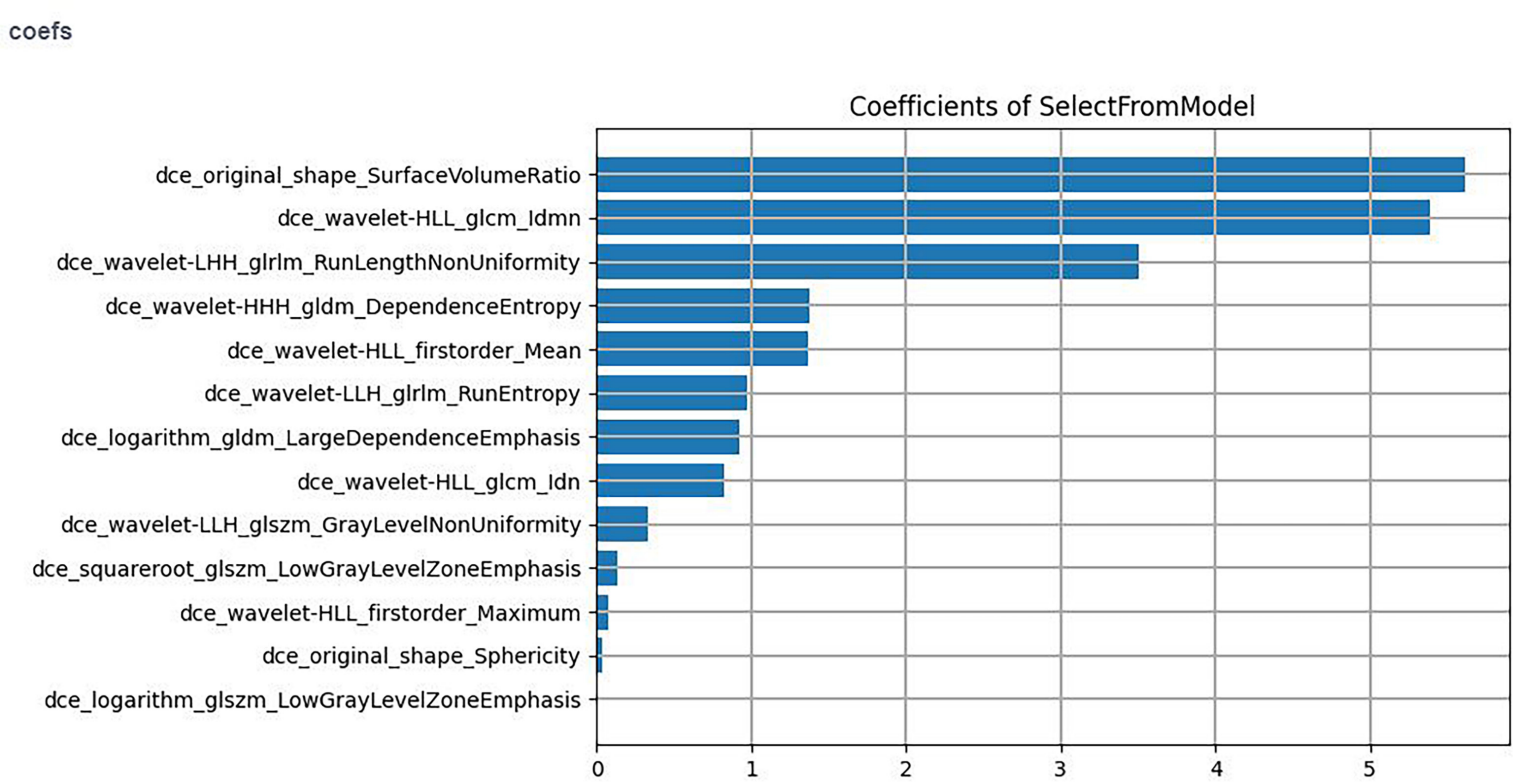
The final selected feature.

The computer-generated datasets were randomly assigned 70% of datasets to the training set (56 positive SLN, 67 negative SLN) and 30% of datasets to the validation set (25 positive SLN, 29 negative SLN). Feature selection plays an important role in training classifiers, reducing computational complexity, and improving classification accuracy. The optimal feature filter (i.e., sample variance F value) was used to evaluate the linear correlation between each feature and category label, and 132 most relevant features were screened out from 1,316 features. LASSO Logistic regression method was used to further select the optimal predictive features from the above features, and 13 features were obtained finally that were most relevant to the prediction of sentinel lymph node metastasis (**Figure 2**), including two first-order statistical features, two shape features, and nine texture features.

### Construction, Validation, and Performance of Machine Learning Model

Five machine learning models, including Support Vector Machine (SVM), Random Forest (RF), Logistic Regression (LR), Gradient Boosting Decision Tree (GBDT), and Decision Tree (DT), were constructed using the optimal feature subsets selected by dimensionality reduction. The predictive performance of five models was further tested in the independent validation set using the same thresholds determined in the training set. The 10-fold cross-validation method was used to verify the accuracy of the models. The receiver operating characteristic (ROC) curve and area under the curve (AUC) were used to evaluate the performance of the above models, and the accuracy, sensitivity, and specificity were calculated.

### Statistical Analysis

The normally distributed variables were shown as mean ± SD, and the skewed variables were shown as median (interquartile range). The independent *t*-test was used to compare the age. Mann-Whitney U-test was used to compare the tumor size. Chi-square cross-tabulation or Fisher’s exact test was used to compare the differences in categorical variables (BI-RADS classification, histological type, histological grade, and molecular subtype). DeLong’s test was used to compare the differences of AUC between five machine learning models. *P*-values < 0.05 were considered statistically significant. DeLong’s test was carried out in R Studio software (version 1.3.1093); other statistical tests were conducted in Statistical Package for Social Sciences (SPSS) software version 22.0 (SPSS Inc., Chicago, IL, USA).

## Results

### Clinical and Histopathological Characteristics

The result of clinical and histopathological characteristics of patients in the training set, validation set, SLN-metastasis group, and non-SLN-metastasis group in the training set are shown in **Table 1**. The clinical and pathological variables between the SLN-metastasis group (*n* = 56 patients) and the non-SLN-metastasis group (*n* = 67patients) in the training set had no significant differences (P > 0.05), except tumor size (P<0.01) and BI-RADS classification (P=0.006). There were no significant differences in all clinical and histopathological characteristics between the training set and the validation set (P > 0.05).

**Table 1:** Clinical and histopathological characteristics.

Characteristics	Training set (*n* = 123)				Validation set (*n* = 54)	P*-value
	Total (*n* = 123)	SLN+ (*n* = 56)	SLN- (*n* = 67)	*P*-value		
Age, years (Mean ± SD)	46.30 ± 10.80	45.07 ± 11.14	47.33 ± 10.48	0.250	46.54 ± 9.60	0.890
Tumor size on MRI, median (IQR), cm	2.20 (1.70–3.10)	2.75 (1.92–3.77)	1.90 (1.50–2.70)	<0.01	2.15 (1.60–2.92)	0.488
**BI-RADS classification**				0.006		0.321
BI-RADS 4	51 (41.5%)	17 (30.3%)	34 (50.7%)		29 (53.7%)	
BI-RADS 5	49 (39.8%)	31 (55.4%)	18 (26.9%)		17 (31.5%)	
BI-RADS 6	23 (18.7%)	8 (14.3%)	15 (22.4%)		8 (14.8%)	
**Histological type**				0. 180		0.259
Invasive ductal carcinoma	114 (92.7%)	54 (96.4%)	60 (89.6%)		47 (87.0%)	
Others	9 (7.3%)	2 (3.6%)	7 (10.4%)		7 (13.0%)	
**Histological grade**				0.689		0.909
I	7 (5.7%)	4 (7.1%)	3 (4.5%)		4 (7.4%)	
II	77 (62.6%)	33 (59.0%)	44 (65.7%)		33 (61.1%)	
III	39 (31.7%)	19 (33.9%)	20 (29.8%)		17 (31.5%)	
**Molecular subtype**				0.418		0.536
Luminal A	20 (16.3%)	7 (12.5%)	13 (19.4%)		11 (20.4%)	
Luminal B	60 (48.8%)	26 (46.43%)	34 (50.8%)		29 (53.7)	
HER-2 positive	18 (14.6%)	11 (19.7%)	7 (10.4%)		4 (7.4%)	
Triple negative	25 (20.3%)	12 (21.4%)	13 (19.4%)		10 (18.5%)	

SLN+, patients with SLN metastasis; SLN−, patients without SLN metastasis; SD, standard deviation; IQR, interquartile range; HER2, human epidermal growth factor receptor-2.

Data are numbers of patients, with percentages in parentheses.

*P*-value < 0.05 indicates a significant difference between SLN+ and SLN− group in the training set.

P*-value < 0.05 indicates a significant difference between training and validation sets.

### Prediction Performance of Machine Learning Models

**Figure 3:**
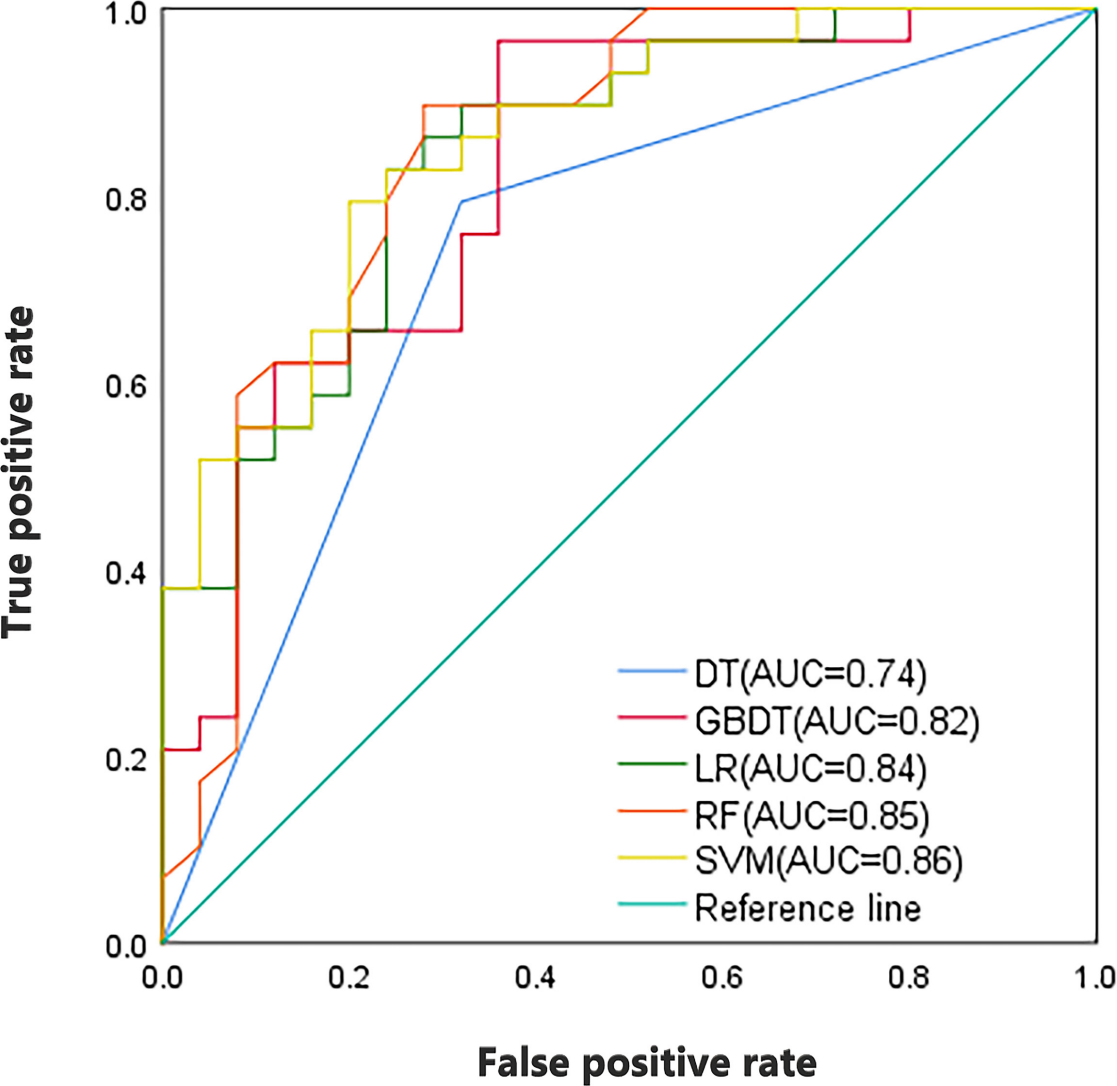
ROC curves of the SVM, RF, LR, GBDT, and DT classifiers in validation set.

As shown in **Table 2**, in the training set, the AUC of SVM, RF, LR, GBDT, and DT were 0.91, 1.00, 0.92, 1.00, and 1.00, respectively. In the validation set, the AUC of SVM, RF, LR, GBDT, and DT were 0.86, 0.85, 0.84, 0.82, and 0.74, respectively. **Figure 3** shows the ROC curves of the five machine learning models. The results of the DeLong test showed that in the validation set, the difference between the AUC of DT and other machine learning models was statistically significant (P<0.05). There was no statistically significant difference in AUC among the four models of SVM, RF, LR, and GBDT (all P>0.05). It can be seen that the decision tree is less effective in predicting axillary lymph node metastasis of breast cancer, while other machine learning models have higher predictive effectiveness. The AUC of SVM was the highest in the validation set, which is 0.86.

**Table 2:** Prediction performance in training and validation sets of five machine learning models.

Machine learning algorithm	Training set				Validation set			
	ACC	SEN	SPE	AUC	ACC	SEN	SPE	AUC
SVM	0.83	0.71	0.94	0.91	0.78	0.60	0.90	0.86
RF	1.00	1.00	1.00	1.00	0.81	0.72	0.90	0.85
LR	0.84	0.77	0.90	0.92	0.78	0.68	0.86	0.84
GBDT	1.00	1.00	1.00	1.00	0.72	0.68	0.76	0.82
DT	1.00	1.00	1.00	1.00	0.74	0.68	0.79	0.74

SVM, support vector machine; RF, Random Forest; LR, logistic regression; GBDT, Gradient Boosting Decision Tree; DT, Decision Tree; ACC, accuracy; SEN, sensitivity; SPE, specificity; AUC, area under the curve.

### Construction, Validation, and Performance of Combined Model

In the univariate analysis, presented in **Table 1**, tumor size and BI-RADS classification were found to be associated with SLN status. To develop a more precise and clinically applicable model to predict an individual’s SLN status, we used the SVM algorithm to construct a combined model incorporating CE-MRI radiomic features, tumor size, and BI-RADS classification. ROC and AUC were used to evaluate the performance of the above models.

**Figure 4:**
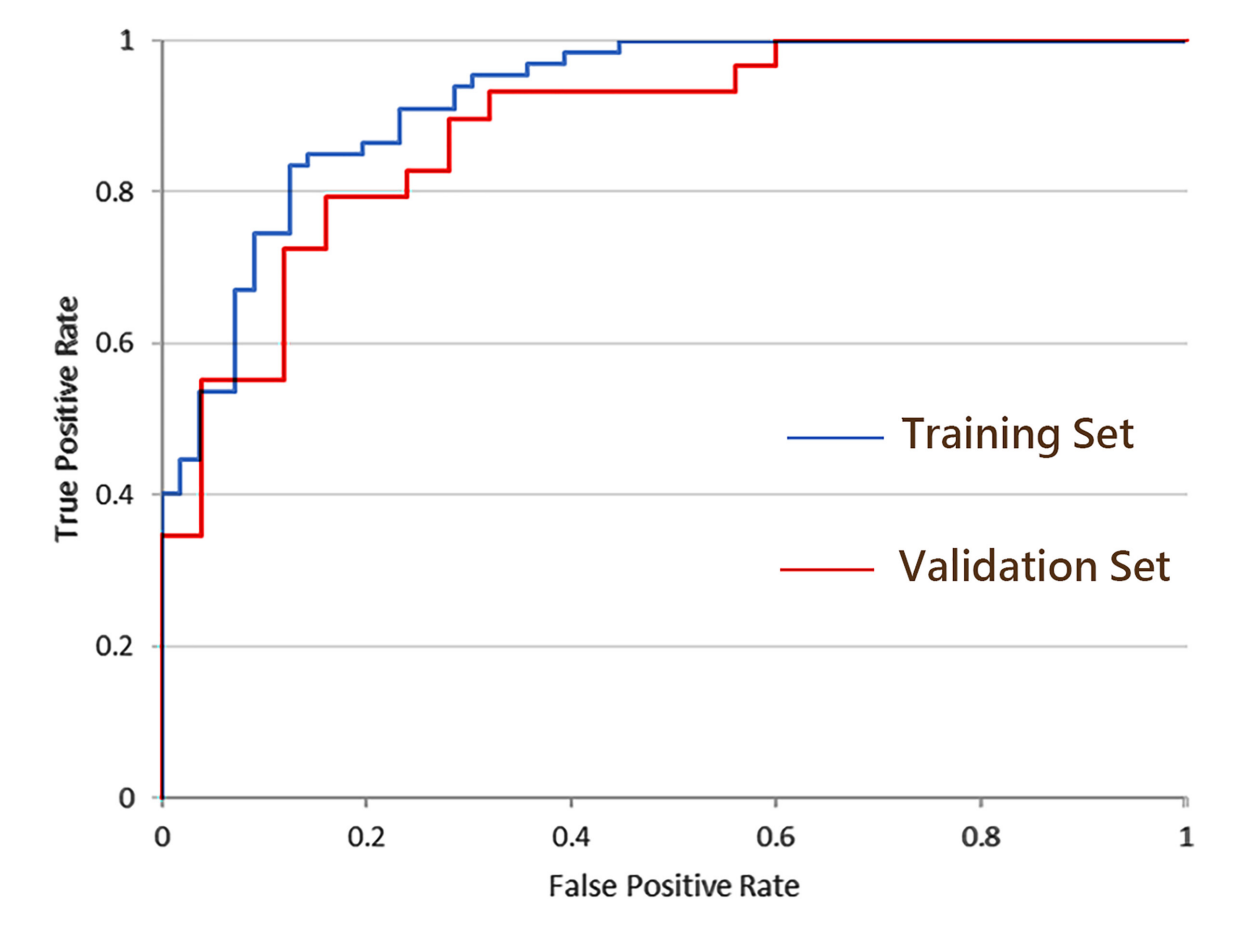
ROC curves of the combined model incorporating CE-MRI radiomics features, tumor size, and BI-RADS classification.

As shown in **Table 3**, the combined model showed better performance in SLNM prediction and achieved a higher AUC in the training set (AUC, 0.92) (**Figure 3**) and the validation set (AUC, 0.88) (**Figure 4**). The combination of CE-MRI radiomic features, clinical characteristics, and BI-RADS classification could improve the predictive ability.

**Table 3:** Prediction performance of training and validation sets of the combined model (SVM algorithm).

Group	ACC	SEN	SPE	AUC
Training set	0.83	0.73	0.91	0.92
Validation set	0.80	0.68	0.90	0.88

SVM, support vector machine; ACC, accuracy; SEN, sensitivity; SPE, specificity; AUC, area under the curve.

## Discussion

With the continuous improvement of comprehensive treatment and deepening of breast cancer research, the surgery of breast cancer is gradually developing towards minimally invasive operation. SLN status is an essential factor in the individualized treatment plan for patients with breast cancer. SLNB is currently the gold standard for determining SLN status in patients with clinically lymph node negative breast cancer. It can also provide details of metastases such as micro-metastasis or macro-metastasis, telling us if patients need further axillary dissection or radiotherapy. However, this approach is invasive and still has some significant limitations. Therefore, how to use the biological information of the primary tumor to predict SLNM highly accurately and non-invasive preoperatively needs to be solved urgently.

In this study, we preliminarily discussed the value of machine learning model based on the radiomic features of CE-MRI in the preoperative prediction of SLNM in breast cancer. This is a non-invasive, fast, and convenient method. The study results showed that SVM, RF, LR, and GBDT models had high AUC values in the validation set. DT is prone to overfitting; the AUC value of its validation set is lower than other machine learning models. Among the four machine learning models other than DT, the AUC (0.86) of SVM was the highest in the validation set. SVM can seek the best compromise between the complexity and learning ability of the model to obtain the best generalization capability based on the limited sample information. It shows many unique advantages in solving small samples, high dimensions, non-linearity, and so on (15, 16). Therefore, we used the SVM algorithm to construct a combined model incorporating CE-MRI radiomic features, tumor size, and BI-RADS classification, and the AUC of its validation set reached 0.88, which outperformed the prediction model using CE-MRI radiomics features alone (AUC=0.86). This suggests that it may provide a high-precision preoperative diagnostic method for automated evaluation of SLNM in clinical practice.

In recent years, the potential predictive efficacy of radiomics in tumor diagnosis, staging, molecular expression, lymph node metastasis, prognosis, and curative effect prediction has been demonstrated in many studies (17–20). Machine learning plays an important role in radiomic analysis, but the predictive performance of different machine learning algorithms is different. To obtain an optimal prediction model, some scholars (21, 22) have compared the prediction performance of different machine learning algorithms. Liu et al. (21) previously established three machine learning models (SVM, LR, and XGBoost) based on the radiomic features of DCE-MRI for the prediction of SLNM in breast cancer patients. SVM has the best predictive performance, with the validation set AUC as high as 0.83, which is slightly lower than the SVM, RF, and LR model in this study. The main reason may be that Liu et al.’s small number of patients leads to poor model training. In addition, Cui et al. (22) showed that the SVM classifier was significantly better than the KNN classifier and LDA classifier in predicting axillary lymph node metastasis of breast cancer, with an AUC of 0.8615, which was similar to the results of this study. All the above experiments used the radiomic features extracted by DCE-MRI to build different machine learning models, and achieved high predictive performance, which indicates that the radiomic features extracted from DCE-MRI of primary tumors may be related to SLNM and reflect the heterogeneity and aggressiveness of breast cancer.

Breast cancer is a kind of tumor with high temporal and spatial heterogeneity. It is often difficult to obtain comprehensive information about the tumor in a timely and effective manner. However, with the rise of radiomics, the radiomic features have gradually come into our field of vision. Many subtle changes in medical imaging are difficult to be observed by the naked eye, but they can be presented through the radiomic features, so as to characterize and explain the subtle changes in tumor biology and provide timely and effective tumor information for clinical practice. Therefore, the selection and extraction of radiomic features also play a significant role in radiomic analysis. At present, many scholars (17, 23–25) have applied ultrasonography, mammography, MRI, and PET-CT in radiomics studies of lymph node metastasis in breast cancer. Among them, MRI is the most commonly used because of its superior temporal and spatial resolution. For example, Liu et al. (26) used DCE-MRI intra- and peritumoral radiomic features to predict ALN metastasis, and the validation cohort AUC was 0.806. However, it is difficult to unify the peritumoral region of each tumor on images, so the extraction of peritumoral features needs further study and verification. Dong et al. (27) combined the radiomic features of FS-T2WI and DWI sequence to predict SLNM in breast cancer patients, and the validation cohort AUC was 0.805, which was lower than the results in this study. Reflected from the side, CE-MRI images may be better than DWI and FS-T2WI images to provide microscopic information of the tumor, indicating intratumor heterogeneity.

In this study, our predictive performance was superior to the previous models that predicted SLN metastasis in breast cancer based only on the clinicopathological features of the primary tumor. Previous studies (28–31) have shown that tumor size, multifocality, histologic tumor type, lymphovascular space invasion, ER status, PR status, and HER-2 status can be independent predictors of breast cancer with SLNM. However, the highest AUC of the prediction model established by these clinicopathological factors was only 0.81, lower than the prediction model based on radiomic features alone established in this study. Therefore, some scholars (17, 32, 33) combined radiomic features with clinicopathological features to study lymph node metastasis of breast cancer. Yu et al. established a clinical-radiomic nomogram model using contrast-enhanced T1-weighted imaging (T1+C), T2WI, and DWI-ADC radiomic features combined with clinicopathological features, and obtained an AUC of 0.90, which was higher than the predictive efficiency of the combined model in this study, while the prediction efficiency of other scholars’ models is lower than this study. The combined model established in this study is simpler than the model established by Yu et al. and has high predictive performance, providing a more convenient and feasible prediction model for clinical practice.

It is worth noting that radiomics, like other techniques, has some technical defects and limitations (34), such as susceptibility toward image acquisition settings, reconstruction algorithms, and image processing. In addition, differences in ROI segmentation, feature extraction, and feature selection will affect the final results of radiomics. The dataset for this study was acquired from a single MR scanner with a consistent scanning protocol, which may minimize confounding factors and potential bias in the extraction and analysis of radiomic features. This study used CE-MRI with high resolution and strictly adhered to a standardized radiomics research process, which will improve the reproducibility of the radiomic features and the stability of the findings in this study. Our study had several limitations. First, this is a preliminary exploratory study with relatively small samples, requiring a larger sample size for further study. Second, this is a retrospective study using a single institutional dataset, lacking generalization and robustness evaluation of our results. Larger studies with more different patient cohorts and imaging datasets merit further investigation. Third, the ROI segmentation of the tumor was not automatically performed, which is time-consuming, error-prone, and user variability. This might be overcome in the future by an automated segmentation artificially intelligent system.

In conclusion, in this study, five machine learning models were established based on CE-MRI radiomic features, revealing the clinical value of machine learning algorithms, and the optimal machine learning algorithm was used to establish a combined model incorporating CE-MRI radiomic features, tumor size, and BI-RADS classification, providing a highly accurate, non-invasive, and convenient method to the preoperative prediction of SLNM in breast cancer patients. Future studies will improve the prediction model further and conduct a multicenter validation study with larger samples.

## Data Availability Statement

All datasets generated for this study are included in the article/**Supplementary Material**.

## Ethics Statement

The studies involving human participants were reviewed and approved by the Institutional Review Board of the First Affiliated Hospital of Soochow University. Written informed consent for participation was not required for this study in accordance with the national legislation and the institutional requirements.

## Author Contributions

LY and YZ created the study design. YZ collected the data and processed the data. YZ and HS conducted data analysis. YZ and LY wrote the manuscript. All authors contributed to the article and approved the submitted version.

## Conflict of Interest

The authors declare that the research was conducted in the absence of any commercial or financial relationships that could be construed as a potential conflict of interest.

## Publisher’s Note

All claims expressed in this article are solely those of the authors and do not necessarily represent those of their affiliated organizations, or those of the publisher, the editors and the reviewers. Any product that may be evaluated in this article, or claim that may be made by its manufacturer, is not guaranteed or endorsed by the publisher.
